# Pathogenesis and Genomic Analysis of a Virulent Leptospira Interrogans Serovar Copenhageni Isolated from a Dog with Lethal Infection

**DOI:** 10.3390/tropicalmed7110333

**Published:** 2022-10-28

**Authors:** Natasha Rodrigues de Oliveira, Frederico Schmitt Kremer, Risciela Salardi Alves de Brito, Rosimeri Zamboni, Odir Antônio Dellagostin, Sérgio Jorge

**Affiliations:** 1 Centro de Desenvolvimento Tecnológico, Núcleo de Biotecnologia, Universidade Federal de Pelotas, Pelotas 96160-000, RS, Brazil; 2 Faculdade de Veterinária, Universidade Federal de Pelotas, Pelotas 96160-000, RS, Brazil

**Keywords:** canine leptospirosis, infection, jaundice, bioinformatics, whole-genome sequencing

## Abstract

Dogs are highly susceptible to leptospirosis and are a public health concern due to their important role as a source of spreading disease, particularly in urban settings. In this study, we present the pathogenesis, serological characterization, and complete genome sequencing of a virulent Brazilian strain (NEG7) of *L. interrogans* serovar Copenhageni isolated from the urine of a dog that died due to acute leptospirosis. Clinical investigation showed that the dog was presented with icteric mucous membranes, weakness, dehydration, anorexia, and kidney and liver failures. Necropsy followed by histopathological evaluation revealed lesions compatible with liver and kidney leptospirosis. The leptospires recovered from the urine were further characterized by genome analysis, which confirmed that the isolate belonged to *L. interrogans* serogroup icterohaemorrhagiae serovar Copenhageni. Multiple bioinformatics tools were used to characterize the genomic features, and comparisons with other available Copenhageni strains were performed. Characterization based on absence of an INDEL in the gene lic12008, associated with phylogenetic and ANI (99.99% identity) analyses, confirmed the genetic relatedness of the isolate with *L. interrogans* serovar Copenhageni. A better understanding of the diversity of the pathogenic Leptospira isolates could help in identifying genotypes responsible for severe infections. Moreover, it can be used to develop control and prevention strategies for Leptospira serovars associated with particular animal reservoirs.

## 1. Introduction

Leptospirosis is a zoonotic disease of global importance that originates from infection caused by the spirochete bacterium, Leptospira [1]. Although bacteria of Leptospira genus have a broad geographical distribution, tropical and subtropical countries with impoverished steel populations carry the greatest disease burden [2,3,4]. The exposure risk is increased by the rapid growth of urban slums owing to favorable conditions for rat-borne transmission [5]. In urban settings, rodents are the main reservoirs for these bacteria, and they shed the pathogen in their urine, thereby contaminating the environment [6,7].

The disease has a complex and dynamic epidemiology [8], with the Leptospira genus comprising the most genetically and antigenically diverse group of spirochetes [9]. Currently, 64 species, 17 of which are genomically classified as pathogenic, with the potential to infect and cause severe disease in humans and a broad range of animal species [10]. Animal leptospirosis represents a risk of infection due to the human-animal-ecosystem interface, which has been a great concern due to its impact on animal health and the following economic losses [11,12]. 

Pathogenic species of the genus Leptospira live in the kidneys of different mammalian species and are excreted through urine. Dogs are at high risk of exposure to leptospires due to constant contact with infected water sources and food exposed to contaminated water or potentially infected by rodents [13]. Serovars belonging to *L. interrogans* serogroups canicola and icterohaemorrhagiae are the most frequently associated strains with canine leptospirosis [14]. Before its importance to public health, the identification and characterization of Leptospira pathogenic strains had a main role in epidemiological investigation of disease dissemination, as dogs may serve as sentinels and indicators of environmental contamination [15]. In view of potential risk involving direct transmission of the Leptospira from dogs to its owners [16], some researchers have also proposed that dogs can act not only as sentinels, but also as possible reservoirs, i.e., “vectors,” establishing a closer contact of wildlife leptospirosis with humans [17,18]. In the present study, we describe the clinical and pathological findings of the acute manifestations of canine leptospirosis. The virulent strain was isolated from urine sample and serological and genomic analyses were performed. The results showed that *L. interrogans* serovar Copenhageni strain NEG7 is a virulent and pathogenic strain. 

## 2. Materials and Methods

### 2.1. Clinical Case

A 10-year-old, male dog, undefined race, weighing 9 kg, unvaccinated against leptospirosis, presented to the Veterinary Clinic in Rio Grande do Sul State, Brazil (31°46′ S, 52°20′ W), with a history of dehydration, pyrexia, shivering, muscle weakness, vomiting, and anorexia detected approximately 3 days earlier. Additionally, the owner related the dog’s exposure to food contaminated by rodents, followed by a rat-bite between 1 and 2 weeks before the veterinary consultation. Physical examination revealed jaundice of the mucosa. Blood was collected via cephalic vein puncture with an anticoagulant for hematological examination, including hematocrit (Ht) and complete blood count (CBC). Blood collected without anticoagulant was used for biochemical and serological examinations, including alanine aminotransferase (ALT), alkaline phosphatase (ALP), blood urea nitrogen (BUN), and creatinine (CR) serum concentrations. EDTA-anticoagulated whole blood and urine samples were collected aseptically for bacterial isolation and urinalysis, respectively. Leptospirosis was treated with both supportive and specific therapies. However, the patient died.

### 2.2. Isolation of Leptospires

Urine and blood samples were collected and immediately inoculated into individual culture media diluted to 10−1, 10−2, and 10−3. Approximately, 500 µL of each dilution was inoculated into tubes containing 4.5 mL of Ellinghausen–McCullough–Johnson–Harris (EMJH) medium containing 200 µg/mL 5-fluorouracil (Sigma-Aldrich, São Paulo, SP, Brazil) to inhibit contaminants and was supplemented with Leptospira Enrichment EMJH (Difco, BD, São Paulo, SP, Brazil). Cultures were incubated at 30 °C and examined weekly using dark-field microscopy for 2–4 months.

### 2.3. Specific Antibodies Detection 

The microscopic agglutination test (MAT) was performed to detect the presence of antibodies in canine serum samples against Leptospira spp. using the following serovars: Autumnalis, Bratislava, Canicola, Copenhageni, Hebdomadis, Grippotyphosa, Hardjo-type hardjo-bovis, Hardjo-prajitno, Icterohaemorrhagiae, Javanica, Pomona, Tarassovi, and Wolffi, as previously described [19]. The MAT titer was expressed as the reciprocal of the highest serum dilution that resulted in 50% agglutination of leptospires. Leptospirosis exposure was defined as MAT ≥100.

### 2.4. Molecular Analysis

For molecular characterization, DNA from the isolated strain was extracted using the Illustra Bacterium GenomicPrep Mini Spin Kit following the manufacturer’s instructions (GE Healthcare, Contagem, MG, Brazil). Seven discriminatory primers were used for VNTR analysis: VNTR4, VNTR7, VNTR9, VNTR10, VNTR11, VNTR19, and VNTR23 [20]. PCR reactions were performed using a cycle of 94 °C for 5 min, followed by 35 cycles of amplification at 94 °C for 30 s, 55 °C for 30 s, 72 °C for 1 min, and a final extension at 72 °C for 7 min. Aliquots were evaluated by agarose gel electrophoresis and the size of the amplified products was estimated by comparison. DNA from the previously characterized *L. interrogans* serovar Copenhageni strain Fiocruz L1-130 was used as the positive control.

### 2.5. Whole-Genome Sequencing

Whole-genome sequencing was performed by next-generation sequencing using an Illumina platform with paired-end libraries (2 × 150 bp). The RAW reads generated were then converted to FASTQ format using the FastQC tool (https://www.bioinformatics.babraham.ac.uk/projects/fastqc/ (accessed on 1 February 2022)). The Trimmomatic tool was used to trim and crop the FASTQ data and remove adapters. De novo assembly was performed using four distinct assemblers: Abyss [21], Ray [22], SPAdes [23], and Velvet [24]. The assemblers’ results were merged using CISA [25]. The statistics of the consensus assembly were generated using QUAST [26]. The final assembly was annotated using the Prokka tool [27] with the default settings.

The full genome was submitted to the NCBI annotation platform [28]. For the identification of Leptospira species, in silico multilocus sequence typing (MLST) analysis was performed using BLASTn searches against a database of alleles (https://github.com/tseemann/mlst (accessed on 1 February 2022)). Sequence types (STs) were assigned using the Leptospira MLST scheme 1 developed by Boonsilp et al. [29], which includes seven housekeeping genes: tpiA, glmU, mreA, pntA, sucA, caiB, and pfkB. The allelic profile and ST obtained were compared with other publicly available sequences from PubMLST (http://pubmlst.org/leptospira (accessed on 1 February 2022)). 

### 2.6. Comparative and Phylogenetic Analysis

The final assembly of the NEG7 genome was aligned with the reference strain *L. interrogans* serovar Copenhageni strain FDAARGOS_203 (GenBank: GCA_002073495.2) using BLASTn from the NCBI BLAST + package [30], and the synteny regions were analyzed using the Artemis comparison tool [31]. To confirm and define species delineation, the average nucleotide identity (ANI) was determined using the FastANI tool [32].

Multiple genome alignment was also performed using Sibeliaz [33] to determine the syntenic regions between the NEG7 strain, 17 pathogenic Leptospira spp. strains, 114 *L. interrogans* serovar Copenhageni strains, and 13 *L. interrogans* serovar Icterohaemorrhagiae strains, all of which are available in the NCBI RefSeq database (Supplementary Data; Table S1). Leptospira core genes derived from PubMLST were searched using NCBI-BLAST + regions on all strains, individually aligned using MUSCLE, and concatenated. Only genes with hits for all strains were used. A maximum likelihood tree was constructed using RaxML and manually annotated using iToL. 

To understand the plasticity of the *L. interrogans* genome, we also compared two other strains of *L. interrogans* serovar Copenhageni from the same sequence type (ST:17): The final assembly of the genome of *L. interrogans* strain NEG7 was aligned to the genome of the virulent *L. interrogans* serovar Copenhageni L1-130 and *L. interrogans* serovar Copenhageni strain M20, a reference strain commonly used in MAT analysis. It was conducted using the Artemis Comparison Tool (ACT) to identify structural variations (e.g., translocations, large insertions/deletions, and inversions). 

Moreover, a list of confirmed pathogenesis-related protein sequences of the pathogenic Leptospira was generated based on literature and previously published data. Pathogenesis-related genes were mapped based on BLASTp analysis to the reference genome sequence of *L. interrogans* serovar Copenhageni strain Fiocruz L1–130 (GenBank: AE016823, AE016824). Antibiotic resistance genes were identified using ResFinder [34] and CARD [35], whereas prophages were identified using PHASTER [36]. 

## 3. Results

### 3.1. Hematological and Serodiagnosis

Hematological abnormalities included neutrophilic leukocytosis, lymphocytosis, eosinopenia, thrombocytopenia, and hypoalbuminemia. Biochemical findings showed that ALAT, bilirubin, creatinine, alkaline phosphatase, phosphorus, and BUN exceeded the reference values. No marked changes in erythrocyte counts were observed, while leukocyte counts exceeded the reference range (data not shown). MAT results showed antibody titers of 100 for serovar Copenhageni and 400 for serovars Canicola and Icterohaemorrhagiae.

### 3.2. Macroscopic and Microscopic Findings

**Figure 1:**
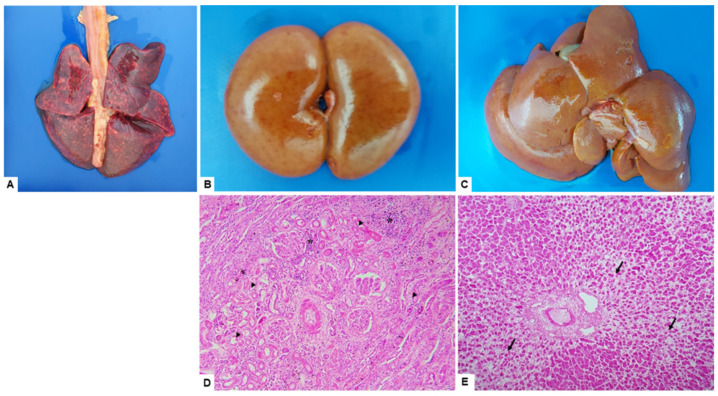
Common gross and histopathological findings of the infected dog. Necropsy findings included generalized icterus, petechial to ecchymotic hemorrhages in the lungs (**A**), kidneys (**B**), and liver (**C**). For histopathological analysis tissues were stained with hematoxylin and eosin (HE). Note, kidneys showing interstitial nephritis with multifocal lymphohistiocytic (asterisk) with diffuse tubular degeneration (arrowhead) (**D**), and liver with massive dystrabeculaton (loss of cohesion) of hepatocytes with degeneration areas, and necrosis (arrows) (**E**). The histopathology photomicrographs were taken at a magnification of ×10.

Macroscopic findings included marked icterus in the subcutaneous tissue, presence of serosanguineous peritoneal fluid, liver with accentuated lobular pattern, kidneys with petechial subcapsular hemorrhages, hemopericardium, pulmonary congestion, hemorrhage, and edema. At the microscopic level, the lungs showed congestion and hemorrhage with intra-alveolar edema, and the kidneys were presented with interstitial nephritis with multifocal lymphohistiocytic and diffuse tubular degeneration. Liver microscopy revealed marked massive hepatocellular dissociation with degeneration and presence of necrotic areas (Figure 1). Additionally, urinary bladder submucosal hemorrhage was observed. Warthin-Starry staining indicated the presence of spirochetes in the tubular cells and the renal interstitium (data not shown).

### 3.3. Isolation and Genomic Analysis

**Figure 2:**
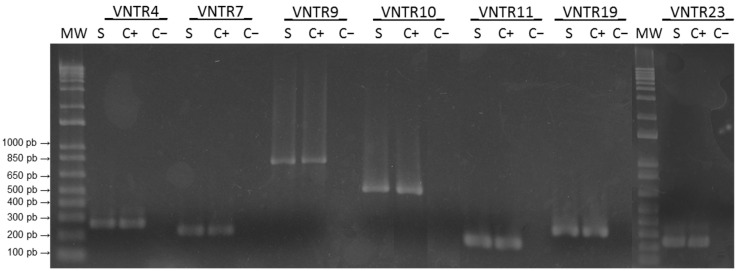
PCR analysis of the polymorphism of seven representative VNTR loci. MW: Molecular weight marker (1 Kb plus DNA ladder). (S) Sample from genomic DNA from Leptospira isolate (named NEG7); (C+) positive control (genomic DNA of *L. interrogans* serovar Copenhageni strain Fiocruz L1-130) and (C−) negative control (water template).

The isolate was successfully cultured from urine but not from blood, indicating bacteremia occurrence only during acute infection. VNTR results revealed the presence of tandem repeats for the seven discriminatory primers used, and the amplicons presented an identical pattern to *L. interrogans* serovar Copenhageni strain L1-130 (positive control). According to the dendrogram proposed by Majed et al. (2005) [20], this pattern identified the species as *L. interrogans*, serogroup Icterohaemorrhagiae serovar Icterohaemorrhagiae/Copenhageni (Figure 2), which is usually indistinguishable by molecular analysis.

Moreover, whole-genome sequencing was performed. The sequenced reads of the strain NEG7 were assembled into 60 contigs with an N50 value of 134,612 (134 Mb). In the final assembly, the genome had a total size of 4.64 Mb, with an average GC content of 35.13% (Table 1). The annotation results identified 3.756 predicted coding sequences (CDS), 39 tRNAs, 6 rRNAs, and 1 tmRNA. MLST analysis revealed that strain NEG7 belonged to sequence type 17 (ST17) of the MLST 1 scheme (Supplementary data; Table S2), which consists of mainly *L. interrogans* strains belonging to serovars Icterohaemorrhagiae and Copenhageni, recovered from humans, dogs, and other animal hosts.

The NEG7 strain showed an ANI of 99.99% with the *L. interrogans* serovar Copenhageni strain FDAARGOS_203 (GCF_002073495.2), as the most closely-related strain. To obtain better serovar discrimination based on genome analysis, lic12008 was tested for the presence of a frameshift mutation, which is present only in Icterohaemorrhagiae strains. The INDEL was absent in the NEG7 isolate, supporting the genetic relatedness of the NEG7 strain to Copenhageni serovars.

**Figure 3:**
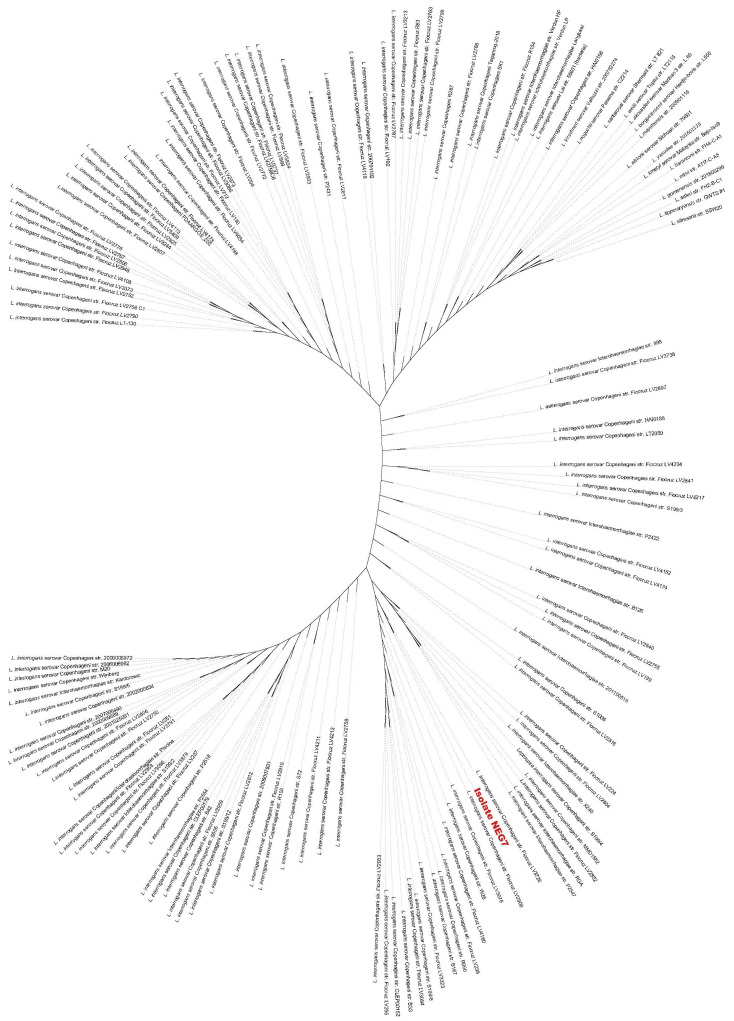
Maximum likelihood tree constructed based on the alignment of the syntenic regions identified as shared by different genomes of *L. interrogans* serovar Copenhageni and Icterohaemorrhagiae strains.

For phylogenetic analysis, the 17 available strains of the pathogenic Leptospira spp. (P1) described by Vincent et al. (2019) [37], 114 *L. interrogans* serovar Copenhageni strains, and 13 *L. interrogans* serovar Icterohaemorrhagiae strains isolated from infected humans and animals were used (the complete list is presented in Supplementary data; Table S1). All strains are available at the NCBI RefSeq. The analysis was based on Leptospira spp. core genes derived from PubMLST, and the results revealed the diversity of the Copenhageni strains. Isolate NEG7 was clustered into a distinct subclade containing only Copenhageni strains, showing close genetic relatedness (Figure 3). Icterohaemorrhagiae strains were dispersed within all subclades, but in most of them, they formed a distinct branch.

**Figure 4:**
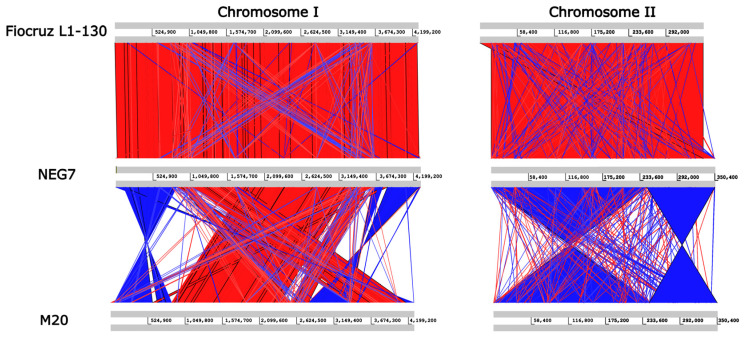
The genome structure rearrangement analysis of the NEG7 strain generated by Artemis comparison tool (ACT), using the genomes of *L. interrogans* serovar Copenhageni strains Fiocruz L1-130 and M20 as reference. Red bands depict similar regions and blue bands illustrate inversions.

A structural comparison was performed using ACT based on the *L. interrogans* serovar Copenhageni strain Fiocruz L1-130 (virulent) and *L. interrogans* serovar Copenhageni strain M20 reference genomes (Figure 4). All contigs were mapped to the two chromosomes, and no extrachromosomal elements were identified (e.g., phages and plasmids). Structural analysis facilitated the identification of some arrangement variations (blocks of inverted sequences between the genomes and translocations) in the respective chromosomes, with the NEG7 isolate showing a genomic structure similar to the virulent L1-130 strain. When the NEG7 genome was compared to the M20 strain, a large number of genomic inversions and translocations were observed between the pairs of genomes.

All the pathogenesis-related gene sequences of the pathogenic Leptospira spp. generated based on the published data were identified in the NEG7 genome, and showed high conservation (98.15–100%) (Table 2). Most of these genes were previously reported to be virulence factors, including genes coding for proteins related to chemotaxis, flagellar machinery, collagenases, sphingomyelinases, and immunogenic surface-exposed proteins. The whole genome shotgun project has been deposited in GenBank under the accession CP093938.1 (Chromosome I) and CP093939.1 (Chromosome II).

## 4. Discussion

Several factors may affect the clinical presentation of leptospirosis in domestic animals, mainly those that are associated with the host, such as the susceptibility of the infected animal and its immune status at the time of infection, and those associated with the pathogen, such as the virulence and infective dose of the Leptospira serovar [38]. In this study, we describe a thorough clinical, serological, and histopathological investigation of a dog that succumbed to severe icteric leptospirosis. Isolation and Leptospira strain characterization have high epidemiological value, which is important for designing prophylactic approaches and improving disease control measures. Here, a virulent Leptospira sp. was isolated from urine, followed by serological and complete genome characterization.

Clinical signs of leptospirosis are usually non-specific. However, in clinical practice, unvaccinated dogs with icterus and/or signs of acute renal failure should be considered suspected cases of leptospirosis. The clinical signs and hematological and biochemical alterations observed in the canine patient from the day of admission were compatible with most clinical manifestations observed in Leptospira infected dogs with jaundice and renal, hepatic, and pulmonary failure. As observed in other dogs with fatal outcomes, serum creatinine and urea levels are high [39]. Histological findings after dog death confirmed severe lung, kidney, and liver involvement, which has been specifically associated with high mortality in dogs [39,40].

In urban settings, dogs are usually raised as pets, in close contact with humans, and the presence of infected dogs represents a major medical and veterinary problem, offering a potential risk of infection for humans, beyond contributing to the environmental spread of the bacteria [38,41]. In urban areas, dogs are usually exposed to environmental contamination spread by synanthropic rats, such as Norway rats (Rattus norvegicus), which act as classic reservoir hosts of Icterohaemorrhagiae and Copenhageni serovars worldwide [6,42]. In this case, the domestic dog, probably exposed to a high bacterial load coming from rodent urine, which was associated with delayed treatment, could be responsible for the severe disease manifestation, resulting in its death.

Dogs are the main maintenance host to pathogenic Leptospira Canicola strains, and can manifest an acute infection or become asymptomatic carriers of leptospires in the urine [38]. The infection by icterohaemorrhagiae serogroup, such as Copenhageni strains, are known to be incidentals, and consequently virulent to dogs, as well as humans, leading to severe clinical development and often resulting in fatal outcomes [14,38,43,44]. Leptospira interrogans serogroup Icterohaemorrhagiae strains have been described as a prevalent serovar on dogs in different regions through the world [14,44,45,46]. Despite broad variability, the main representative serovars from this serogroup are Icterohaemorrhagiae and Copenhageni strains, which show similar clinical, serological, and genomic characteristics.

Seroepidemiological and genomic investigations conducted in Brazil have shown evidence from Icterohaemorrhagiae as the main infecting serogroup found in stray, symptomatic, and asymptomatic dog populations [47,48], with Copenhageni serovar appearing as the main circulating serovar [49]. Ref. [46] isolated 12 strains of pathogenic *L. interrogans* serogroup Icterohaemorrhagiae from blood and urine samples of naturally infected dogs. In a study performed by [44], the researchers found molecular evidence for the presence of the Copenhageni L1-130 strain in the urine of asymptomatic dogs from a shelter in São Paulo. Cunha et al. (2022) [50] also found seropositivity to Copenhageni serovar in 62.5% of dogs from households in a major city in southern Brazil.

Until recently, Icterohaemorrhagiae and Copenhageni serovars could not be differentiated by molecular techniques and genome sequencing, requiring characterization using the MAT technique with different panels of hyperimmune sera and mAbs for the suspicious serovars [20,51]. Serovar typing is performed only by a few reference laboratories [52], which hampers the precise identification of Leptospira clinical isolates in routine research. Santos et al. (2018) [53] reported the occurrence of a frameshift mutation within a homopolymeric tract of lic12008 gene, which is present only in *L. interrogans* serovar Icterohaemorrhagiae strains and absent in Copenhageni strains. This INDEL shows high discriminatory power, allowing discrimination of Icterohaemorrhagiae from Copenhageni serovars by sequencing the lic12008 gene from Leptospira strains isolated from different geographic locations and hosts.

In this study, genome sequencing revealed that lic12008 INDEL was absent in the genome of our isolate NEG7, which, associated with ANI and phylogenetic analysis results, indicated that the isolate belonged to the Copenhageni serovar. Chromosomal rearrangement and phylogenetic analysis of the NEG7 strain showed a close genetic relationship with the virulent Copenhageni L1-130. Additionally, whole-genome sequencing of the NEG7 isolate showed that this strain harbored almost all the confirmed pathogenesis-related genes in Leptospira spp., with a high level of conservation.

## 5. Conclusions

This study presented the complete clinical statement, agent isolation, genome sequence data, and features of a highly virulent strain of Leptospira serovar Copenhageni isolated from a convalescent dog with leptospirosis. The availability of clinical, pathological, and especially genomic information regarding the infective virulent strains is valuable and can add information useful for further studies aiming to better understand the occurrence of virulence factors that drive leptospirosis transmission and establishment of lethal infections in incidental hosts. Furthermore, these data might help in identifying better options for infection prevention measures and contribute to the epidemiological characterization of common circulating strains among dogs.

## Figures and Tables

**Table 1:** Overview of the genome characteristics for the final assembly of the *L. interrogans* strain NEG7 genome.

Assembly							
Size (Mb)	CG%	#Contigs	N50 (pb)	CDSs ^a^	tRNAs	rRNAs	tmRNA
4.64	35.13	60	134,612	3756	39	6	1

^a^ Coding DNA sequences.

**Table 2:** Pathogenesis-related genes identified in the genome of *L. interrogans* strain NEG7 using BLAST.

Gene	Function	NEG7 Locus Tag	L1-130 Locus Tag	Identity (%)
*ClpB*	Molecular chaperone-Resist heat and oxidative stress	MPF80_10065	LIC_12017	100.00
*ColA*	Collagenase A-Dissemination	MPF80_13785	LIC_12760	100.00
*FcpA*	Flagella coling protein-Dissemination	MPF80_15850	LIC_13166	100.00
*FlaA2*	Flagella assembly-Dissemination	MPF80_04025	LIC_10787	100.00
*FliM*	Flagella motor switch protein-Dissemination	MPF80_09170	LIC_11836	100.00
*FliN*	Flagella motor switch protein-Dissemination	MPF80_06905	LIC_11370	100.00
*HemO*	Heme oxygenase-Iron acquisition	MPF80_18235	LIC_20148	100.00
*HtpG*	High-temperature protein G-associated with acute disease	MPF80_17695	LIC_20044	100.00
*KatE*	Catalase-Resist oxidative stress	MPF80_10140	LIC_12032	100.00
Unknown	Associated with host colonization	MPF80_14995	LIC_12791	99.74
Unknown	LPS biosynthesis associated with acute disease	MPF80_10675	LIC_12142	100.00
Unknown	Associated with host colonization	MPF80_06245	LIC_11235	98.15
Unknown	Associated with host colonization	MPF80_14995	LIC_20153	100.00
*LenA*	Endostatin-like protein-Adhesion to ECM	MPF80_10440	LIC_12096	100.00
*LenB*	Endostatin-like protein-Adhesion to ECM	MPF80_05070	LIC_10997	100.00
*LenC*	Endostatin-like protein-Adhesion to ECM	MPF80_15100	LIC_13006	100.00
*LenD*	Endostatin-like protein-Adhesion to ECM	MPF80_11560	LIC_12315	100.00
*LenE*	Endostatin-like protein-Adhesion to ECM	MPF80_17370	LIC_13467	100.00
Unknown	Gene regulation	MPF80_18045	LIC_20111	100.00
*LigA*	Immonoglobulin-like repeat protein Adhesion to ECM	MPF80_02420	LIC_10465	100.00
*LigB*	Immonoglobulin-like repeat protein Adhesion to ECM	MPF80_02410	LIC_10464	100.00
*LipL21*	Neutrophil myeloperoxidase inhibitor	MPF80_00115	LIC_10011	100.00
*LipL32*	Most abundant outer-membrane lipoprotein in pathogenic leptospires	MPF80_06820	LIC_11352	100.00
*LipL36*	Abundant outer-membrane lipoprotein	MPF80_15375	LIC_13060	100.00
*LipL41*	TonB-dependent receptor-Iron acquisition	MPF80_14890	LIC_12966	99.72
*LipL45*	Adhesion to ECM	MPF80_08230	LIC_11643	99.74
*Loa22*	Associated with acute disease	MPF80_01045	LIC_10191	100.00
*LpxD1*	Lipid A biosynthesis protein-Temperature adaptation	MPF80_15300	LIC_13046	100.00
*LruA*	Associated with acute disease	MPF80_05095	LIC_11003	100.00
*Lsa21*	Adhesion to ECM; potent TLR2 and TLR4 agonist	MPF80_01940	LIC_10368	100.00
*LvrA*	hybrid histidine kinase-*Leptospira* virulence regulator system	MPF80_08530	LIC_11709	100.00
*LvrB*	hybrid response regulator-*Leptospira* virulence regulator system	MPF80_08525	LIC_11708	100.00
*Mce*	Mammary cell entry protein-Cell entry	MPF80_09280	LIC_11859	100.00
*OmpL1*	OmpA family protein-Adhesion to ECM	MPF80_04940	LIC_10973	100.00
*OmpL37*	Adhesion to ECM	MPF80_11285	LIC_12263	100.00
*OmpL47*	Adhesion to ECM	MPF80_15320	LIC_13050	100.00
*Sph2*	Sphingomyelase 2-Hemolytic activity	MPF80_13140	LIC_12631	100.00
*TlyA*	Hemolysin A-Hemolytic activity	MPF80_01505	LIC_10284	100.00
*TlyC*	Hemolysin-Hemolytic activity	MPF80_15755	LIC_13143	100.00

No resistance genes were identified by ResFinder or CARD RGI. However, four putative prophage regions were found by PHASTER, all located on chromosome I. These regions vary in length from 6.1 to 11.4 Kb and are located in the regions 1395373-1404767, 2566578-2577980, 2652849-2659925, and 3155117-3161281.

## Data Availability

The original contributions presented in the study are included in the article/Supplementary Material. Further inquiries can be directed to the corresponding author.

## Supplementary Materials

The following supporting information can be downloaded at: https://www.mdpi.com/article/10.3390/tropicalmed7110333/s1, Table S1: List of genomes of *Leptospira* strains considered to the phylogenetic analysis; Table S2: MLST analysis for the final assembly of the *L. interrogans* strain NEG7 genome.
